# Dipalmitoylphosphatidic acid inhibits breast cancer growth by suppressing angiogenesis via inhibition of the CUX1/FGF1/HGF signalling pathway

**DOI:** 10.1111/jcmm.13727

**Published:** 2018-07-16

**Authors:** Jian Chen, Zijun Zhou, Yuying Yao, Jianwei Dai, Dalei Zhou, Lijing Wang, Qian‐Qian Zhang

**Affiliations:** ^1^ Vascular Biology Research Institute School of Basic Course Guangdong Pharmaceutical University Guangzhou China; ^2^ GMU‐GIBH Joint School of Life Sciences Guangzhou Medical University Guangzhou China; ^3^ The State Key Lab of Respiratory Disease Guangzhou Institute of Respiratory Disease The First Affiliated Hospital Guangzhou Medical University Guangzhou China

**Keywords:** angiogenesis, breast cancer, CUX1/FGF1/HGF signalling, dipalmitoylphosphatidic acid

## Abstract

Tumour growth depends on a continual supply of the nutrients and oxygen, which are offered by tumour angiogenesis. Our previous study showed that dipalmitoylphosphatidic acid (DPPA), a bioactive phospholipid, inhibits the growth of triple‐negative breast cancer cells. However, its direct effect on angiogenesis remains unknown. Our work showed that DPPA significantly suppressed vascular growth in the chick embryo chorioallantoic membrane (CAM) and yolk sac membrane (YSM) models. Meanwhile, tumour angiogenesis and tumour growth were inhibited by DPPA in the tumour tissues of an experimental breast cancer model, a subcutaneous xenograft mouse model and a genetically engineered spontaneous breast cancer mouse model (MMTV‐PyMT). Furthermore, DPPA directly inhibited the proliferation, migration and tube formation of vascular endothelial cells. The anti‐angiogenic effect of DPPA was regulated by the inhibition of Cut‐like homeobox1 (CUX1), which transcriptionally inhibited fibroblast growth factor 1 (FGF1), leading to the downregulation of hepatocyte growth factor (HGF). This work first demonstrates that DPPA directly inhibits angiogenesis in cancer development. Our previous work along with this study suggest that DPPA functions as an anti‐tumour therapeutic drug that inhibits angiogenesis.

## 1 INTRODUCTION

Angiogenesis refers to the process by which cells sprout to form capillaries and neovasculature from pre‐existing mature endothelial cells of the blood vessels wall.^1^ An efficient supply of nutrients and oxygen via angiogenesis is necessary for tumour cell proliferation and helps tumour cells enter into the circulation and further metastasize to distant organs.^2^ Efficient anti‐angiogenic strategies have emerged as a new modality to treat cancers via the inhibition of pro‐angiogenic molecules. Vascular endothelial growth factor (VEGF), fibroblast growth factor 1 (FGF) and hepatocyte growth factor (HGF) are primary growth factors that promote angiogenesis.^3^ Currently, most inhibitors for the anti‐angiogenic treatment of advanced cancer in clinical trials mainly target VEGF or its receptors (VEGFR).^3^ However, the performance of these agents is still disappointing. Therefore, a deeper exploration of anti‐angiogenic agents is urgently required. Breast cancer is a heterogeneous, multi‐factorial disease. Progression is associated with pathological angiogenic changes in most breast cancers. Within the past decades, angiogenesis has become widely accepted to be essential for breast cancer cell proliferation and metastasis.^4^ Moreover, angiogenesis is considered as a potential prognostic indicator in primary breast cancer.^5^ Recently, the combination of anti‐angiogenesis agents with chemotherapy regiments was used to treat metastatic breast cancer in the clinic. However, a single agent of anti‐angiogenic treatment is reported to be limited in breast cancer therapy.^6^ The angiogenic characteristics are distinct among the different molecular subtypes of breast cancer. Therefore, the clinical benefits of anti‐angiogenic therapies might differ among the different breast cancer subtypes. More effective anti‐angiogenic agents to treat one or more subsets of breast cancers should be further explored.

Dipalmitoylphosphatidic acid (DPPA) is an important intermediate metabolite that is mainly generated from glycerol phospholipid via hydrolysis by phospholipase D (PLD). DPPA plays a critical role in regulating drug penetration into the cells through the cell membrane.^7^ Recently, the function of DPPA in the regulation of pathological changes has begun to be clarified. Recently reports indicated that DPPA plays a critical role in regulating Bcl‐2 expression in a cell type‐dependent manner.^8^, ^9^ In addition, DPPA reportedly inhibits renal interstitial fibrosis in a rat diabetic nephropathy model.^10^ Our previous research demonstrated that DPPA inhibited tumour growth in triple‐negative breast cancer (TNBC), a basal‐like subtype breast cancer.^11^ Meanwhile, we showed that DPPA also decreased angiogenesis in tumour tissue.^11^ However, whether DPPA plays a direct or indirect role in angiogenesis requires further validation.

The transcription factor Cut‐like homeobox1 (CUX1), also known as CUTL1, CDP or Cut, is involved in the regulation of tumour cell proliferation, cell cycle progression, migration, invasion and apoptosis.^12^, ^13^, ^14^ The function of CUX1 is cell type‐dependent, and it acts as a transcriptional activator or transcriptional repressor in different types of cells. High CUX1 expression in pancreatic neuroendocrine tumour cells reportedly promotes angiogenesis via a paracrine pathway.^15^ However, the directly autocrine regulatory role and transcriptional regulatory mechanism of CUX1 in angiogenesis is still unknown.

To identify the direct effect of DPPA, which is involved in angiogenesis, we performed vascular growth analysis using the chick embryo CAM and YSM models. In these assays, the inhibition of blood vessel formation was obviously observed in the DPPA‐treated embryos compared with that in the dimethyl sulphoxide (DMSO)‐treated embryos. Based on these results, we aimed to characterize the effect of DPPA on controlling tumour angiogenesis in different subtypes of breast cancer. Here, we demonstrated that DPPA strongly inhibited tumour growth and angiogenesis in a variety of breast cancer tumour model systems in vivo. Meanwhile, we demonstrated an unequivocal inhibitory effect of DPPA on the activity of growth, migration and tube formation in vascular endothelial cells. In addition, we investigated the related signalling pathways involved in DPPA‐induced anti‐angiogenesis. Here we demonstrated that CUX1 stimulated the transcription of FGF1, thereby further mediating HGF expression. Moreover, the important role of CUX1/FGF1/HGF signalling in promoting cell proliferation, migration and tube formation in vascular endothelial cells was strongly associated with the DPPA‐induced inhibition of tumour growth and angiogenesis in breast cancer.

## 2 MATERIALS AND METHODS

### 2.1 Reagents and antibodies

Dipalmitoylphosphatidic acid (BML‐LP103) was obtained from Enzo Life Sciences, Inc. (NY, USA) and was dissolved in DMSO at the concentration of 10 mmol/L according to our previously reported.^11^ Cell Counting Kit‐8 (CCK8) was obtained from Beyotime (Shanghai, China). Rabbit anti‐GAPDH (#2118) was obtained from Cell Signaling Technology, Inc. (USA), rabbit anti‐CD31 (ab28364) was obtained from Abcam (Cambridge, MA), rabbit anti‐FGF1 (BA0843) and rabbit anti‐HGF (BA0911) were obtained from Boster (Wuhan) Co., Ltd. (China), and rabbit anti‐CDP (sc‐13024) was obtained from Santa Cruz Biotechnology Inc. (Santa Cruz, CA, USA). All siRNAs were synthesized by RiboBio Co., Ltd. (Guangzhou, China).

### 2.2 Cell lines and transfection

Primary human umbilical vein vascular endothelium cells (HUVEC, ATCC^®^ PCS‐100‐010™) were obtained from ATCC and maintained in endothelial cell growth medium including growth supplements (EGM, CC‐3124, Lonza). The MCF‐7 luminal‐like breast cancer cell line was obtained from the cell bank of the Chinese Academy of Sciences (Shanghai, China) and maintained in Dulbecco's Modified Eagle's Medium (DMEM) with high glucose supplemented with 100 U/mL of penicillin, 100 μg/mL streptomycin and 10% foetal bovine serum (FBS). The cells were incubated at 37°C in a humidified incubator supplemented with 5% CO_2_. All the siRNAs were transfected into HUVECs using Lipofectamine 3000 (Invitrogen) at a final concentration of 100 nmol/L.

### 2.3 Animals and treatment

The spontaneous breast cancer MMTV‐PyMT mice (stock no: 002374) were obtained from the Jackson Laboratory (Bar Harbor, Maine, USA) and the BALB/c athymic nude mice (5 weeks old, male) were obtained from the Guangdong Medical Laboratory Animal Center. All mice were housed in a temperature‐ and humidity‐controlled room with a 12‐hour light‐dark cycle. All the experiments were performed according to protocols that approved by the Center of Laboratory Animals Ethics Committee of Guangdong Pharmaceutical University.

The BALB/c athymic nude mice were inoculated subcutaneously with MCF‐7 cells (10^6^ cells) at the second right mammary fat pad area to construct a subcutaneous xenograft tumour model and randomly divided into two groups. The mice were then treated with DPPA (3 mg/kg bodyweight) or DMSO at equal concentrations once every 2 days for 10 days via an intravenous tail injection at 7 days after the tumour cell inoculation. The female MMTV‐PyMT mice (8 weeks old) were also divided into two groups and treated with DPPA or DMSO for 4 weeks as indicated above. The length (L) and width (W) of the tumours were measured with calipers and the tumour volume (V) was calculated as V = (L × W^2^) × 0.5236.

### 2.4 Chick embryo chorioallantoic membrane (CAM) assay

The fertilized chicken eggs were obtained from the Avian Farm of South China Agriculture University (Guangzhou, China). The egg shells were cleaned with 75% ethanol and incubated in a temperature‐ and humidity‐controlled incubator at 37 ± 1°C and 50%‐60% humidity. After 9 days, an approximately 1 × 1 cm window was cut above the air chamber. Then, DMSO or DPPA (2, 4 or 8 μmol/L) was added to the air chamber of the live chick embryos and incubated for an additional 48 hours. The CAM vasculature was photographed under a stereomicroscope (Olympus SZX16), and the micro‐vessel density (MVD) was analyzed as the percentage of blood vessel area among the total area.

### 2.5 Chick embryo yolk sac membrane (YSM) assay

The fertilized chicken eggs were incubated in a temperature‐ and humidity‐controlled incubator for 3 days. After incubation, the well‐developed live eggs were carefully cracked into sterile dishes. A colour marked silastic ring was carefully displaced onto the top of the vessel regions in the yolk sac membrane. Then, DMSO or DPPA (2, 4 or 8 μmol/L) was added into the centre of the silastic rings, and they were incubated for 24 hours. The MVD was photographed and quantified at 0, 12 and 24 hours.

### 2.6 Experimental breast cancer assay

The fertilized chicken eggs were incubated for 10 days and prepared as described above for the CAM assay. MCF‐7 cells (10^7^ cells) or MDA‐MB‐231 cells (5 × 10^6^ cells) were added in the middle of the silastic rings, which were placed on the CAM and incubated for 48 hours. Then, DMSO or DPPA (2, 4 or 8 μmol/L) was added into the centre of the silastic rings and incubation for an additional 48 hours. The tumour volume and MVD were further calculated after the tumours were peeled out from the silastic ring.

### 2.7 Cell viability assay

HUVECs (1500 cells/well) and MCF‐7 cells (4000 cells/well) were plated in 96‐well plates, and DPPA and DMSO were added at the indicated concentrations 24 hours later. Forty‐eight hours post‐treatment, the CCK8 reagent was added to the drug‐treated cells, and they were further incubated at 37°C for 3 hours. Following the incubation, the absorbance was evaluated at 450 nm with a plate spectrophotometer.

### 2.8 In vitro HUVEC cell migration assay

HUVECs pre‐treated with DMSO or DPPA were harvested and re‐suspended in EBM medium without growth supplements. The DPPA or a corresponding dose of DMSO was added to the cell suspension, which was placed in the upper compartment of the chamber and in the well that contained EGM with growth supplements. Twelve hours post‐incubation, the chambers were stained with crystal violet for 15 minutes, and cells on the upper surface of the chamber were then carefully removed with a cotton swab. The membranes were photographed and the number of migrated cells was calculated under an inverted light microscope.

### 2.9 In vitro HUVEC tube formation assay

HUVECs were treated with the indicated concentration of DPPA or DMSO for 48 hours. Then, the tube formation assay was carried out as previously reported.^16^ The pre‐treated HUVECs were harvested and added to a 96‐well plate, which was pre‐coated with Matrigel, at a density of 3 × 10^4^ cells/well. Meanwhile, the cells were further treated with DPPA or DMSO and maintained at 37°C for 5 hours to form a vascular tube. The tubes were photographed and the length of the tubes was measured using an inverted light microscope.

### 2.10 Real‐time quantitative PCR (qRT‐PCR) arrays

Total RNA was extracted from the cells treated with DMSO or DPPA using the TRIzol reagent (Invitrogen). Total RNA was reverse transcribed and real‐time quantitative PCR analysis was carried out using an RT^2^ first strand kit (#330401, Qiagen) and RT² Profiler™ PCR Array Human Angiogenic Growth Factors (PAHS‐072Z, Qiagen) according to the manufacturer's instructions. The fold change of RNAs in the DPPA group compared with the DMSO group were determined, and the genes were altered up to 5‐fold in the DPPA group over the DMSO group was reported.

### 2.11 Histological and immunoblotting analyses

The tumour tissues were peeled off from the CAM model or mouse models and fixed, embedded and cut into sections. For the histological analysis, the 4‐μm‐thick sections were stained with hematoxylin& eosin (H&E) or incubated with the relevant primary antibodies for immunohistochemical (IHC) and immunofluorescence (IF) assays according to a previously reported.^17^ The total proteins from the tumour tissues and cells were extracted using RIPA buffer. Then, the proteins were separated using SDS‐polyacrylamide gel electrophoresis (PAGE) and transferred onto polyvinylidene fluoride (PVDF) membranes. The membranes were incubated with the relevant antibodies and further visualized by exposure to the Image Quant LAS 4000 system (GE Healthcare).

### 2.12 Statistical analysis

All data are expressed as the mean ± standard deviation (SD) of 3 separate experiments. Differences between two groups were analyzed using a Student's two‐sided *t*‐tests. *P* values <.05 (*P *<* *.05) were considered statistically significant. The protein bands were quantified densitometrically and normalized to the expression of GAPDH, which was used as the loading control, using Quantity One software (Bio‐Rad, USA). Protein expression on the IHC slides was quantified by measuring the integrated optical density (IOD) using the IPP image analysis software.

## 3 RESULTS

### 3.1 DPPA inhibits tumour growth in luminal‐like breast cancer mouse models

**Figure 1:**
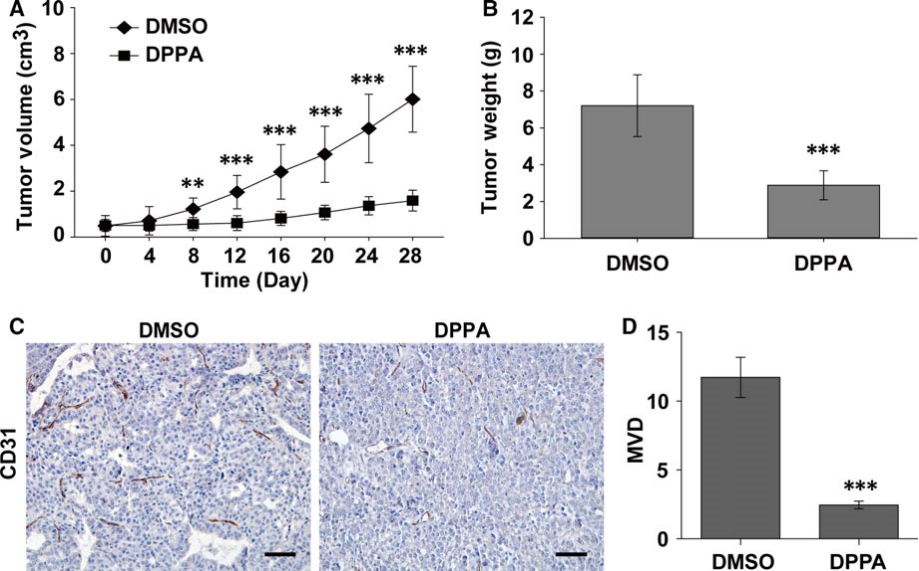
Dipalmitoylphosphatidic acid (DPPA) suppresses tumour growth and angiogenesis in spontaneous luminal‐like breast cancer. A, MMTV‐PyMT mice (8‐wk‐old) were treated with dimethyl sulphoxide (DMSO) or DPPA (3 mg/kg) intraperitoneally once every 2 d for 4 wk, and the tumour volumes were measured every 4 d. DPPA significantly inhibited the tumour volume compared with that in the DMSO group. n = 10, **P <* *.01, ***P <* *.001. B, The tumours were isolated and weighted post‐treatment. DPPA markedly suppressed tumour weight compared with that in the DMSO group. n = 10, ***P <* *.001. C, Immunohistochemical staining for blood vessels using the CD31 vascular endothelial marker on tumour tissues sections. Abundant CD31‐positive was abundant in the DMSO‐treated tumour tissues compared with that in the DPPA‐treated samples. Scale bars 50 μm. D, Statistical analysis showing that DPPA significantly inhibited micro‐vessel density in the tumour tissues compared with that in the DMSO group. ***P <* *.001

In our previous report, we demonstrated that DPPA inhibited tumour growth in TNBC mouse models. However, whether DPPA still possesses anti‐tumour activity in luminal‐like breast cancer remains to be clarified. The MMTV‐PyMT spontaneous breast cancer transgenic mouse is a well‐characterized model to simulate human luminal‐like breast cancer.^18^, ^19^ In this study, MMTV‐PyMT mice and MCF‐7 cells subcutaneous xenograft tumour model mice were employed to elucidate the possible anti‐tumour role of DPPA in luminal‐like breast cancer in vivo. Female MMTV‐PyMT mice were randomly divided into two groups and treated with DPPA (3 mg/kg bodyweight) or the same concentration of DMSO once every 2 days for 4 weeks. The tumour volume was calculated every 4 days, and the tumour volume was significantly reduced by DPPA from the eighth day post‐injection compared to that in the DMSO group (Figure 1A). At the end of the treatment, the mice were sacrificed and the tumours were peeled and weighted. As shown in Figure 1B, the tumour weight was notably decreased under the DPPA treatment compared with those treated with DMSO. Furthermore, tumour angiogenesis was evaluated by staining for CD31, an endothelial cell marker, by an IHC assay. The number of CD31‐positive cells was significantly lower in the DPPA treatment group compared with that in the DMSO group (Figure 1C). In addition, statistical analysis revealed that the MVD was dramatically suppressed by DPPA compared with that in the control group (Figure 1D).

Moreover, the MCF‐7 luminal‐like breast cancer cell subcutaneous xenograft tumour model was constructed and treated with DMSO or DPPA on the seventh day after the cell injection as indicated above. The tumour volume was measured every 2 days, and DPPA significantly inhibited tumour growth from the fourth day of treatment compared with DMSO (Figure S1A). The tumour weight and number of CD31‐positive cells were both decreased by DPPA compared with those in the DMSO group (Figure S1B,C). Moreover, DPPA could notably inhibited the MVD compared with that in the DMSO group (Figure S1D). Together, these data indicated that DPPA inhibits tumour growth and angiogenesis in luminal‐like breast cancer.

### 3.2 DPPA inhibits angiogenesis in vivo

**Figure 2:**
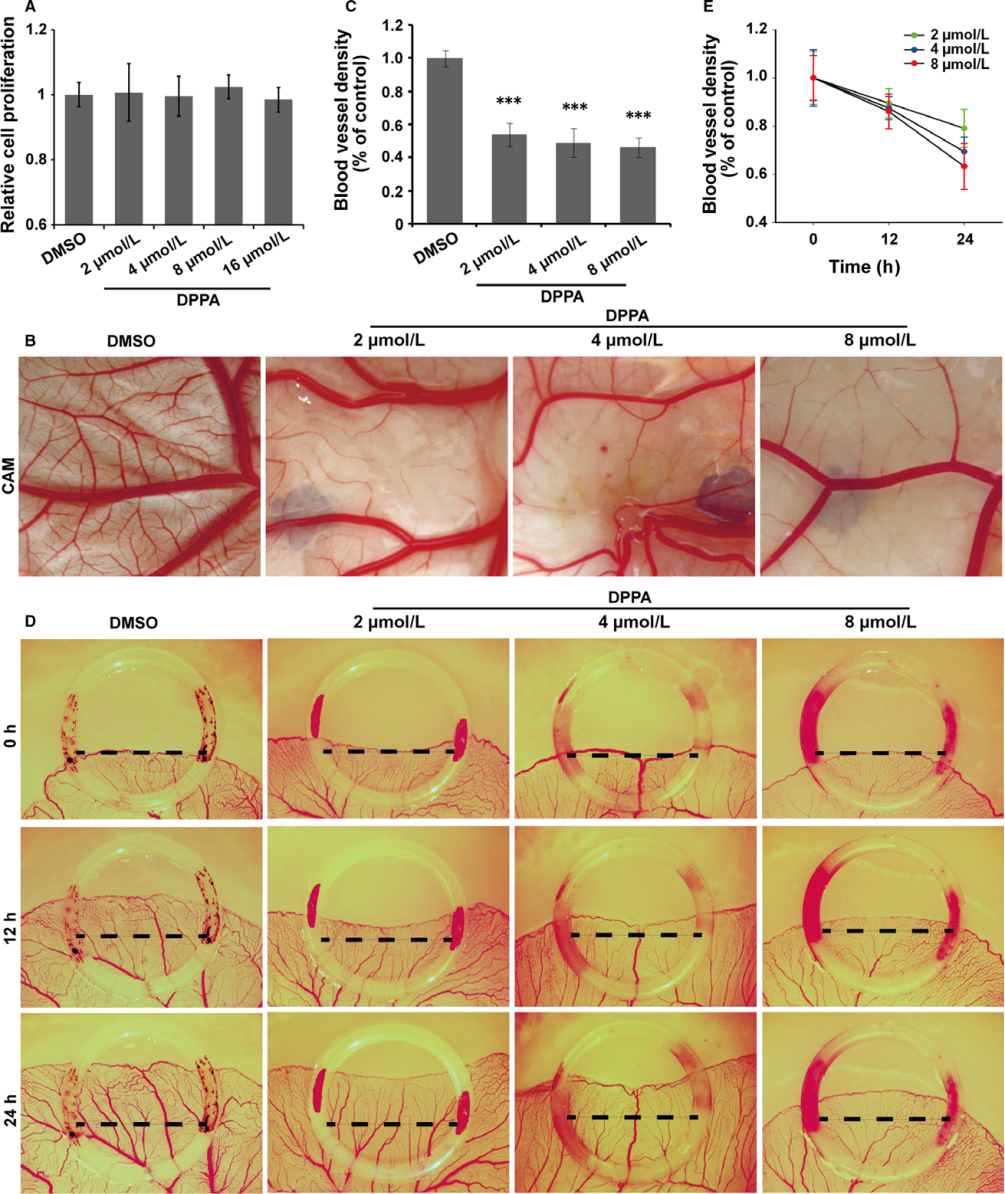
In vivo anti‐angiogenic activity of dipalmitoylphosphatidic acid (DPPA). A, Relative cell proliferation of cells treated with dimethyl sulphoxide (DMSO) or the indicated concentration of DPPA as determined using the CCK8 assay. Cell proliferation was not affected by DPPA at the concentrations of 2, 4, 8 or 16 μmol/L. B, Chick embryo CAM assay. Representative images showing the blood vessels in the CAM model treated with DMSO or DPPA (2, 4 or 8 μmol/L) for 24 h. Fewer blood vessel branches were observed in the CAMs that were DPPA‐treated compared with those in the DMSO‐treated group. C, Statistical analysis of CAMs showing that DPPA significantly inhibited the vascular density in the CAM model compared with that in the DMSO group. Each of the CAM images are representative of 6 independent experiments. ***P <* *.001. D, Chick embryo YSM assay. Representative images of the vascular network in the chick embryo YSM model treated with DMSO or DPPA (2, 4 or 8 μmol/L) at 12 or 24 h post‐treatment. Statistical analysis of the blood vessel density in the YSM treated with DMSO or DPPA. E, Statistical analysis showing that DPPA markedly inhibited the blood vessel plexus. Each YSM image is representative of 6 independent experiments

We demonstrated that a high concentration of DPPA (up to 100 μmol/L) inhibited cell proliferation in TNBC.^11^ To explore the inhibitory role of DPPA on luminal‐like breast cancer cells, MCF‐7 cells were treated with different concentrations of DPPA and DMSO, the final concentrations of DMSO were kept below 0.1% in all the in vitro experiments. Additionally, DPPA was shown to increase Bcl‐2 expression at 10 μmol/L in Hela cells.^8^ Surprisingly, DPPA did not inhibit the proliferation of MCF‐7 cells at the low concentration (Figure 2A). The result was different from that we previously reported in TNBC.^11^ In addition, DPPA reportedly plays an anti‐apoptotic role in a cell type‐dependent manner.^8^, ^9^ Therefore, DPPA may inhibit tumour growth in different subtypes of breast cancer via different regulatory mechanism. The data above indicated that DPPA inhibited vascular density in tumour tissues of luminal‐like breast cancer. We can speculate that one possible mechanism by which DPPA inhibits tumour growth is by blocking angiogenesis in luminal‐like breast cancer.

Accordingly, we further detected the anti‐angiogenic role of DPPA in the early embryonic development model of chicks. The chick embryo CAM and YSM models, which are well‐characterized naturally in culture environments for vascular growth, were employed to study the directly anti‐angiogenic effects of DPPA. The chick embryo CAM was treated with either DMSO or 2, 4 or 8 μmol/L DPPA. After 48 hours of incubation, the angiogenesis and growth of vascular network branches in the CAM were significantly inhibited by DPPA (Figure 2B). In addition, the density of the microvessel plexus in the CAM was markedly suppressed by DPPA (Figure 2C). Moreover, the chick embryo YSM was also treated with DMSO or DPPA, as indicated above, and the blood vessels were photographed at 0, 12 and 24 hours. DPPA significantly inhibited the growth and extension of the blood vessel plexus in the YSM (Figure 2D). The MVD in the YSM was remarkably reduced by DPPA in a dose‐ and time‐dependent manner (Figure 2E). All of these results confirmed that DPPA directly inhibited angiogenesis.

### 3.3 DPPA suppresses the angiogenesis and tumour growth of experimental breast cancer in a chick embryo CAM model

**Figure 3:**
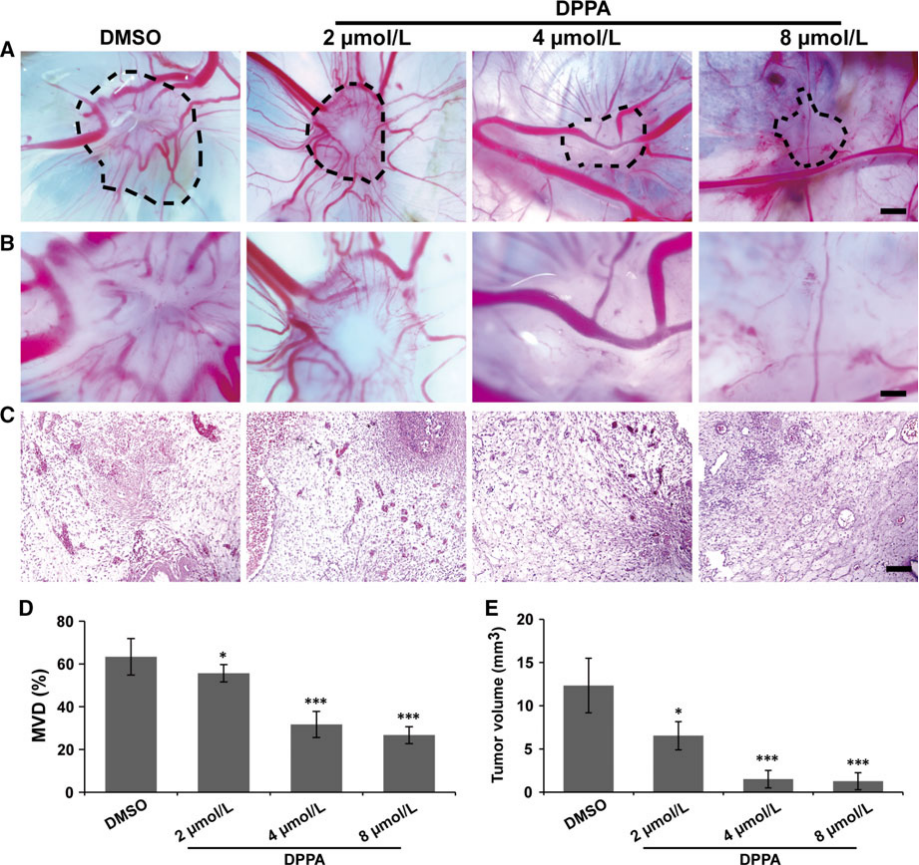
Dipalmitoylphosphatidic acid (DPPA) inhibits angiogenesis and tumour growth in a chick embryo chorioallantoic membrane (CAM) experimental luminal‐like breast cancer model. MCF‐7 cells were seeded on the 10‐d‐old chick embryo CAM through a window in the egg shell and incubated for 48 h, and then treated with either dimethyl sulphoxide (DMSO) or DPPA (2, 4 or 8 μmol/L) for 48 h. A, Representative images of the breast cancer xenografts on CAM treated with DMSO or DPPA (2, 4 or 8 μmol/L). Scale bars, 500 μm. B, Higher magnification of A. Fewer blood vessel plexi were observed in the DPPA‐treated tumour tissues than in the DMSO‐treated group. Scale bars, 100 μm. C, Representative H&E staining of tumour tissues on CAMs. Abundant blood vessels were observed in the DMSO‐treated tumour tissues compared with those in DPPA‐treated tumour tissues. Scale bars, 100 μm. D, Statistical analysis of the microvascular density (MVD) of the DMSO‐ and DPPA‐treated tumour tissues that implanted on CAMs. E, Statistical analysis of the tumour volumes of DMSO‐ and DPPA‐treated tumours that were implanted on CAMs. The tumours in the DPPA‐treated group were much smaller than those in the DMSO‐treated group. *P <* *.05, ***P <* *.001

Tumour cell growth in the chick embryo CAM model depends only on the supply of oxygen and nutrition by blood vessels. Therefore, tumour cells were cultured on the surface of a 10‐day‐old chick embryo CAM model to further investigate the anti‐tumour role of DPPA vis the inhibition of angiogenesis. MCF‐7 cells (luminal A) and MDA‐MB‐231 cells (TNBC) were seeded on the CAM and treated with either DMSO or 2, 4 or 8 μmol/L DPPA. Tumour angiogenesis was notably inhibited by DPPA compared with that inhibited by DMSO in luminal‐like breast cancer (Figure 3A,B) and TNBC (Figure S2A,B). Furthermore, H&E staining showed that the neovascularization in two types of breast cancer tumour tissues was markedly suppressed by DPPA compared with that in the DMSO group (Figure 3C and Figure S2C). In addition, statistical analysis revealed that the MVD was significantly reduced by DPPA in the two types of breast cancer tumour tissues (Figure 3D and Figure S2D). The tumour volume was notably decreased by DPPA compared with that in the DMSO group (Figure 3E and Figure S2E). Because a low concentration of DPPA cannot suppress the cell proliferation of MCF‐7 and MDA‐MB‐231 cells, the inhibition of tumour growth may have resulted from decreased angiogenesis. Together, these results demonstrated that a low concentration of DPPA inhibited angiogenesis and further resulted in the inhibition of tumour growth in both the luminal‐like and TNBC subtypes of breast cancer.

### 3.4 DPPA impedes microvessel formation ex vivo

**Figure 4:**
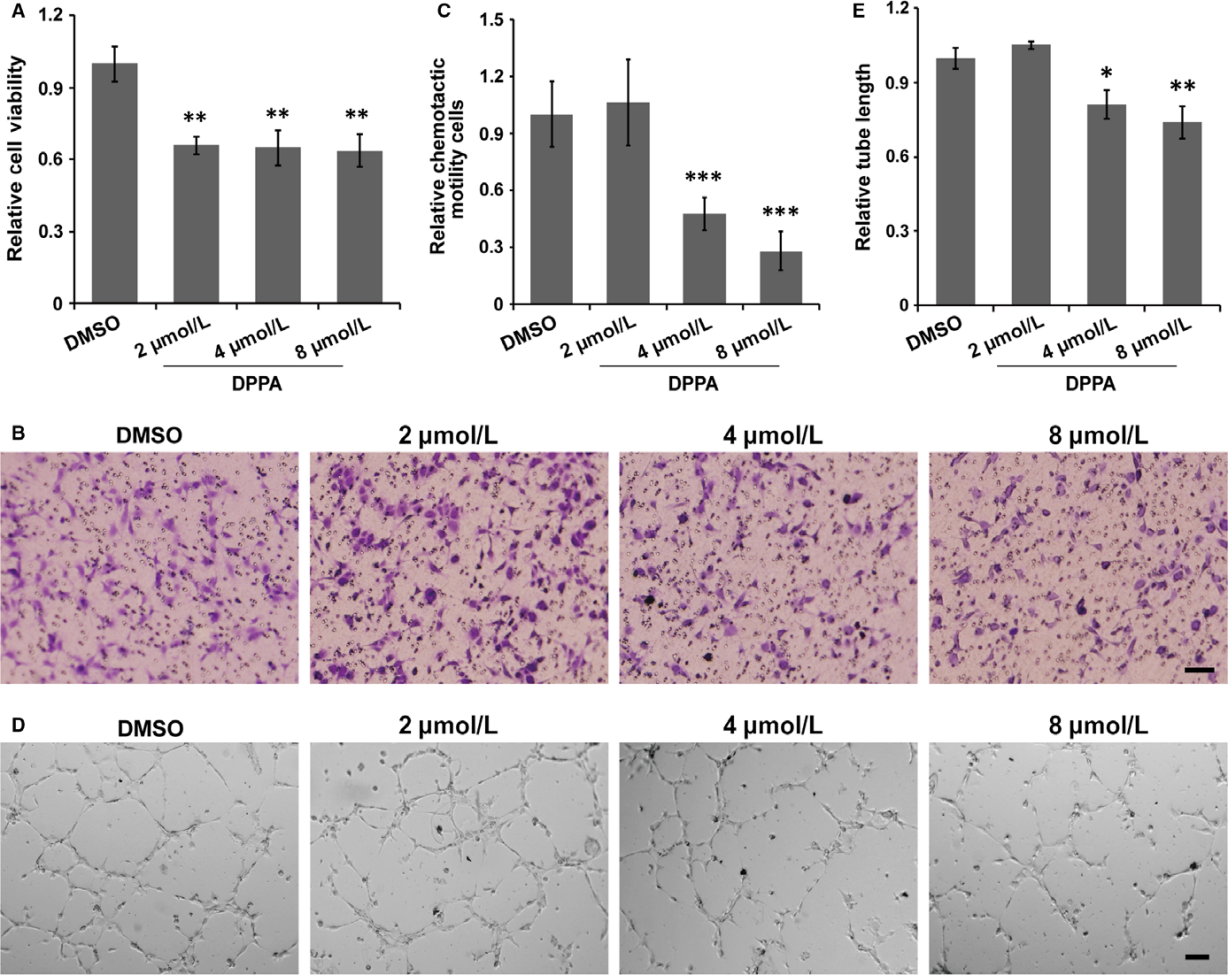
Dipalmitoylphosphatidic acid (DPPA) inhibits the cell proliferation, migration and tube formation of vascular endothelial cells. A, HUVECs were treated with dimethyl sulphoxide (DMSO) or DPPA (2, 4 and 8 μmol/L) for 48 h, and the cell proliferation was measured using the CCK8 assay. DPPA significantly inhibited the proliferation of HUVECs. **P <* *.01. B, The effect of DPPA on the cell migration ability of HUVECs was evaluated using a transwell assay. DMSO or the indicated concentration of DPPA was added to the upper compartment of the chamber, and cells that migrated to the bottom surface of the chamber were then photographed and counted after 20 h of treatment. Scale bars, 100 μm. C, The number of migrated cells were markedly decreased upon DPPA treatment at a concentration >4 μmol/L, ***P <* *.001. D, HUVECs were seeded on Matrigel in the presence of DMSO or the indicated concentration of DPPA, and tube formation was then observed under an inverted microscope at 5 h post‐treatment. Scale bars, 100 μm. E, The DPPA‐treated cells showed significantly decreased the tube formation at a concentration >4 μmol/L, *P <* *.05, **P <* *.01

Angiogenesis is dependent on the cell proliferation, migration and tube formation of vascular endothelial cells. Therefore, the directly anti‐angiogenesis effect of DPPA was further explored in vascular endothelial cells ex vivo. DPPA significantly inhibited cell proliferation in HUVECs at concentrations >2 μmol/L (Figure 4A). Then, the migration of vascular endothelial cells was evaluated, revealing that DPPA markedly suppressed cell migration at concentrations >4 μmol/L (Figure 4B,C). Meanwhile, the tube formation assay showed that HUVECs, treated with DPPA at concentrations >4 μmol/L did not form the capillary‐like microtubule networks compared to those treated with DMSO (Figure 4D,E). Together, these results demonstrated that DPPA inhibited angiogenesis by suppressing the cell proliferation, migration and tube formation ability of vascular endothelial cells.

### 3.5 Involvement of CUX1/FGF1/HGF signalling in DPPA‐mediated angiogenesis inhibition

**Figure 5:**
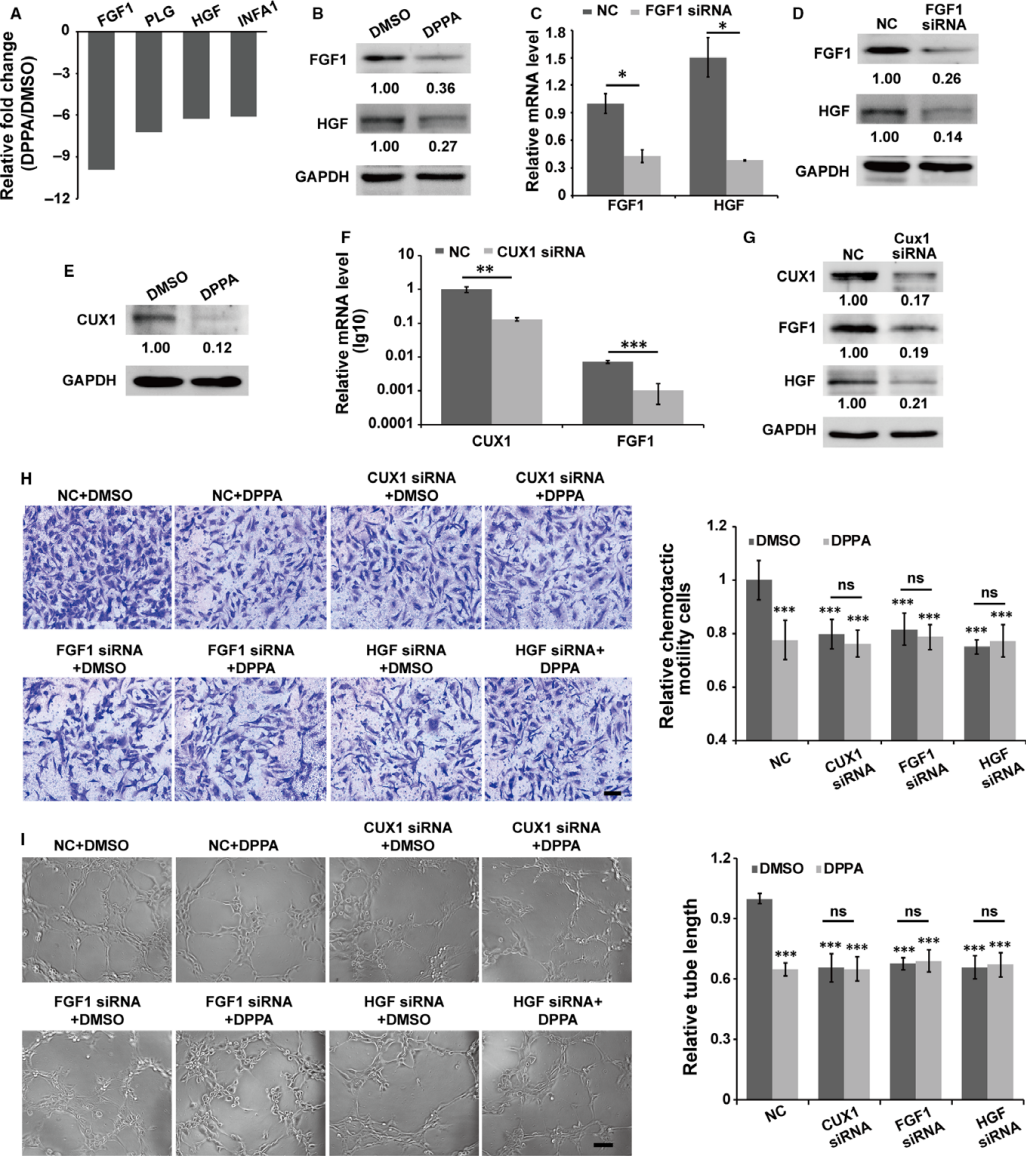
CUX1/FGF1/HGF signalling is involved in dipalmitoylphosphatidic acid (DPPA)‐induced angiogenesis. A, HUVECs were treated with dimethyl sulphoxide (DMSO) or DPPA (8 μmol/L) for 4 h. Then, a human angiogenesis gene array was used to evaluate the potential factors involved in DPPA‐induced angiogenesis. The genes that differential expression more than 5‐fold are shown in the statistical data. B, Proteins from the DMSO‐ or DPPA‐treated HUVECs were extracted and analyzed using an immunoblotting assay. The bands of the immunoblot further confirmed the inhibitory effect of DPPA on FGF1 and HGF expression. Inhibiting FGF1 expression using siRNA reduced the mRNA and protein expression of HGF, as detected by qRT‐PCR and immunoblotting analyses (C,D). *P <* *.05, E, DPPA‐inhibit CUX1 expression was confirmed by immunoblotting analysis. Inhibiting the expression of CUX1 using siRNA reduced the mRNA and protein expression of FGF1, as detected by qRT‐PCR and immunoblotting analyses (F,G). **P <* *.01, ***P <* *.001. HUVECs were transfected with siRNAs for 48 h, and then harvested and treated with DMSO or DPPA to further detected their migration and tube formation abilities. The inhibition of CUX1 or FGF1 or HGF suppressed cell migration and tube formation in HUVECs, and further treatment with DPPA did not further enhance the inhibitory effect of the siRNAs (H,I). Scale bars, 100 μm (G) and 200 μm (H), ns: no significantly difference, ***P <* *.001

Angiogenesis is critically dependent on the action of angiogenic growth factors. However, the regulatory mechanism of DPPA on angiogenesis is still unclear. Therefore, a quantitative RT‐PCR array that included human angiogenic growth factors was used to screen the differential expression angiogenic growth factors associated with the DPPA‐mediated inhibition of angiogenesis. DPPA regulated the differential expression of 4 candidate genes in the array by more than 5‐fold compared to that in the DMSO group (Figure 5A). A report indicated that FGF2 stimulates HGF transcription and protein expression in ischaemic limbs.^20^ FGF1 and FGF2 are both FGF members that are involved in angiogenesis. However, whether FGF1 possesses the same function as FGF2 in the regulation of HGF remains to be demonstrated. DPPA significantly inhibited the expression of FGF1 and HGF (Figure 5B). Meanwhile, the inhibition of FGF1 decreased the mRNA and protein expression of HGF (Figure 5C,D). These results suggested that FGF1 promotes HGF expression and the inhibitory effect of DPPA on angiogenesis might occur via the suppression of FGF1/HGF signalling. However, the regulatory mechanism of DPPA on the inhibition of FGF1 expression needs further confirmation.

One study revealed that FGF1 is transcriptionally regulated by CUX1 in the Hs578T human breast cancer cell line and in the Bon‐1 human pancreatic neuroendocrine tumour cell line.^15^, ^21^ In this study, we explored whether CUX1 is involved in the DPPA‐mediated FGF1 inhibition. DPPA significantly inhibited the expression of CUX1 in HUVECs (Figure 5E). Meanwhile, CUX1 acted as a transcriptional activator, and the inhibition of CUX1 notably reduced the transcription and protein expression of FGF1, subsequently inhibiting HGF expression in HUVECs (Figure 5F,G). Furthermore, inhibiting the expression of CUX1, FGF1 and HGF significantly reduced the migration and tube formation of HUVECs. However, DPPA cannot inhibit the migration and tube formation further, while CUX1/FGF1/HGF signalling was suppressed (Figure 5H,I). Together, these results revealed that CUX1/FGF1/HGF signalling is the main target of DPPA in the inhibition of tumour angiogenesis.

## 4 DISCUSSION

**Figure 6:**
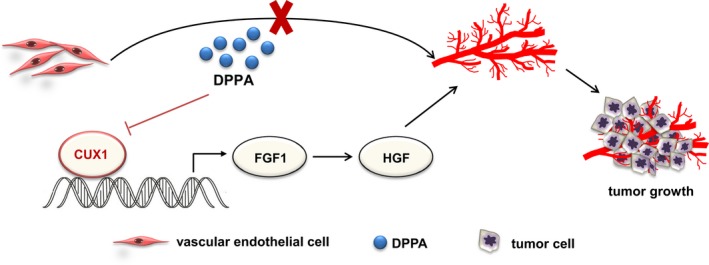
A schematic illustration of how dipalmitoylphosphatidic acid (DPPA) might inhibit angiogenesis in breast cancer. DPPA functions as an anti‐tumour drug in breast cancer by partially inhibiting tumour angiogenesis. The constitutive inhibition of CUX1 by DPPA decreases the mRNA and protein expression of FGF1 and further suppresses HGF expression, which results in the suppression of angiogenesis and abnormal tumour growth in luminal‐like breast cancer and TNBC

In this study, we clearly clarified the role and regulatory mechanism of DPPA on anti‐angiogenesis in breast cancer (Figure 6). Our findings demonstrated that DPPA inhibited angiogenesis during the development of breast cancer. CUX1 was a molecular target of DPPA and transcriptionally activated FGF1. In addition, the inhibition of CUX1 suppressed FGF1 and further decreased the expression of HGF. The targeting of CUX1/FGF1/HGF signalling by DPPA resulted in the inhibition of vascular endothelia cells forming new microvessels.

Dipalmitoylphosphatidic acid is an important minor phospholipid intermediate that is mainly produced by PLD and is widespread in mammalians. However, few studies have examined the regulatory role of DPPA in pathologic processes. In diabetic nephropathy, DPPA acts as an important drug to treat renal interstitial fibrosis by activating AKT signalling.^10^ Recently, DPPA was shown to increase Bcl‐2 expression in Hela cells.^8^ That report suggests that DPPA may exert an anti‐apoptotic effect and promotes tumourigenesis in cervical adenocarcinoma. Nevertheless, DPPA exhibited an anti‐tumour effect on TNBC in our previous study.^11^ As reported in this work, DPPA might also inhibit tumour growth in luminal‐like breast cancer. In addition, we demonstrated that the microvessel density in the tumour tissue sections of TNBC was decreased after DPPA treatment.^11^ In our study, the proliferation, migration and tube formation abilities of HUVECs decreased with DPPA treatment. Together, the findings in our study lead to the conclusion that DPPA directly inhibits angiogenesis. In addition, we further demonstrated that DPPA suppresses breast cancer growth partially by decreasing the vascular density in tumour tissues.

Tumour angiogenesis plays a vital role in tumour growth and progression. More importantly, most tumours cannot growth beyond 2‐3 mm^3^ without the formation of new blood vessels.^22^ Breast cancer is a class of angiogenesis‐dependent tumours, and the prognosis of patients with primary tumours is significantly correlated with the extent of angiogenesis.^5^, ^23^ Therefore, anti‐angiogenic treatment in combination with chemotherapeutic regimens for breast cancer in preclinical or clinical studies has been under investigation for decades. However, the clinical impact has not been very effective until today. Breast cancer can be classified into different molecular subtypes, including luminal‐A, luminal‐B, HER2‐enriched, basal‐like and normal‐like, according to the gene expression pattern.^24^, ^25^ In addition, the angiogenic characteristic and anti‐angiogenic treatment effects are markedly different among these subsets of breast cancers.^23^ Accordingly, the anti‐angiogenesis trials may be patient‐specific in breast cancer therapy. The chick embryo experimental tumour model is a tumour growth model, in which the growth of tumour cells is only supported by only blood vessels. In this study, we employed two subclasses of experimental breast cancer models, luminal‐like and TNBC (a subtype of basal‐like), to explore the anti‐tumour effect via angiogenesis inhibition. We found that a low concentration of DPPA did not inhibit the cell proliferation of breast cancer. However, it did inhibit tumour growth by decreasing the formation of new blood vessels in the chick embryo experimental breast cancer models of luminal‐like breast cancer and TNBC. Our findings first demonstrated that DPPA inhibited tumour growth by suppressing angiogenesis at least in luminal‐like breast cancer and TNBC.

CUX1 is a transcription factor that activates or represses transcription to regulate tumour progression in many cancers.^26^, ^27^ The study of anti‐angiogenesis often focuses on the regulation of angiogenesis‐pathway gene. The overexpression of CUX1 in pancreatic neuroendocrine neoplasm cells upregulates hypoxia inducible factor‐1α (HIF‐1α) and matrix metalloprotein 9 (MMP9), and the paracrine stimulation of endothelial cells then leads to tumour angiogenesis.^15^ However, the biochemical role of CUX1 in vascular endothelial cells renains unclear. In this study, CUX1 promoted the migration and tube formation of HUVECs. Our findings provide important evidence for understanding the role of CUX1 in vascular endothelial cells during the formation of new vessels. Additionally, we further confirmed that CUX1 is the main target of DPPA during the progression of angiogenic inhibition.

As a transcriptional regulator, CUX1 activates or suppresses the expression of multiple genes. However, the genes regulated by CUX1, which are involved in DPPA‐induced anti‐angiogenesis, are still unknown. CUX1 transcriptionally activates the FGF1 gene in Hs578T human breast cancer cells.^21^ However, FGF reveals no difference in response to CUX1 expression in Bon‐1 pancreatic neuroendocrine cancer cells.^15^ To elucidate the DPPA induced anti‐angiogenic phenotype, we assessed a human angiogenesis gene array at the mRNA level using DPPA‐treated HUVECs. In our study, FGF1 was the most significantly decreased gene (more than 5‐fold) in the DPPA‐treated HUVECs compared with that in the DMSO‐treated group. In addition, we also demonstrated that FGF1 is involved in DPPA‐induced anti‐angiogenesis. Our findings further confirmed that CUX1 plays a transcriptional regulatory role that is cell type‐ dependent.

Members of the FGF family, mostly FGF1 and FGF2, exert a pro‐angiogenic effect on endothelial cells.^28^, ^29^ Previous studies showed that FGF2 promotes HGF transcription and protein expression in ischaemic limbs and fibre hyperplasia.^20^, ^30^ However, whether FGF1, which belongs to the FGF family, possesses a similar role in the regulation of HGF is unknown. In our study, we demonstrated that HGF mRNA and protein expression was decreased by decreasing FGF1. These results illustrated that FGF1 exerts the same regulatory function in promoting HGF expression. However, the potential regulatory mechanism still needs further exploration.

In summary, our data successfully demonstrated that DPPA can induce an anti‐angiogenesis effect and further inhibits tumour growth, and DPPA can potentially serve as an anti‐angiogenic therapy drug that is used in luminal‐like breast cancer and TNBC clinical treatments.

## CONFLICT OF INTEREST

The authors have no conflicts of interest to declare.
