# Supplementary material for: Dipalmitoylphosphatidic acid inhibits breast cancer growth by suppressing angiogenesis via inhibition of the CUX1/FGF1/HGF signalling pathway
Source: J Cell Mol Med. 2018 Jul 16;22(10):4760–70. doi: 10.1111/jcmm.13727 (PMC6156235; doi:10.1111/jcmm.13727)

Supplementary Figures

Supplementary Figure Legends

Supplementary Figure 1. DPPA suppresses tumor growth and angiogenesis in xerograph luminal-like breast cancer mice model. (**A**) The MCF-7 cells (10^6 cells) were subcutaneously inoculated in the second right mammary fat pad area of BALB/c athymic nude mice. The mice were treated with DPPA (3 mg/kg body weight) or the same concentration of DMSO once every two days for 10 days through intravenous tail injection at 7 days after cell inoculated. The tumor volume was significantly smaller in DPPA-treated mice. n=8, ***P*<0.01, *** *P*<0.001. (**B**) The tumors were isolated and weighted after treated. Tumor weight was markedly lighter while DPPA treated than DMSO treated. n=8, *** *P*<0.001. (**C**) IHC staining for blood vessels using CD31 on tumor tissues sections. The CD31-positive staining in DMSO treated tumor tissues was more than that in DPPA treated. Scale bars 50 μm. (**D**) The statistical analysis of IHC staining for CD31 showed that DPPA significantly inhibit MVD in tumor tissues. ** *P*<0.01.

Supplementary Figure 2. DPPA inhibits angiogenesis and tumor growth in a chick embryo CAM experimental luminal-like breast cancer model. MDA-MB-231 cells were cultured on the 10-day-old chick embryo CAM and incubated for 48 h, then treated with DMSO or DPPA (2 μM, 4 μM or 8 μM) and further incubated for 48 h. (**A**) Representative images of xenograft tumors on CAM treated with DMSO or DPPA. Scale bars 500 μm. (**B**) Higher magnification of A. Fewer blood vessel plexus in the DPPA-treated tumor tissue than that in the DMSO-treated group. Scale bars 100 μm. (**C**) Representative H&E staining of tumor tissues on CAMs. Abundant blood vessels can be seen in DMSO-treated tumor tissues than that in DPPA-treated tumor tissues. Scale bars 100 μm. Statistical analysis of

microvascular density (MVD) and tumor volume of DMSO- and DPPA-treated tumor tissues that implanted on CAMs (**D and E**). The MVD were decreased and tumors were much smaller in DPPA-treated group than that in DMSO-treated group. **P*<0.05, ** *P*<0.01, *** *P*<0.001.

Supplementary Figure 1

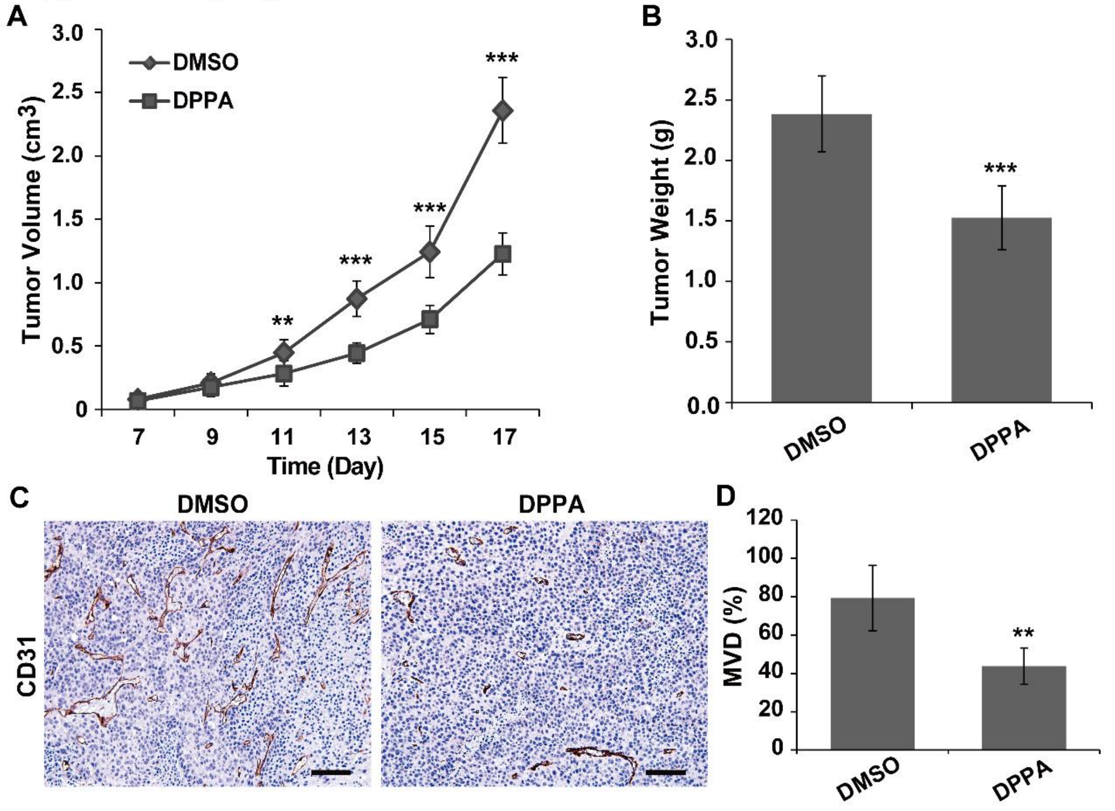

Supplementary Figure 2

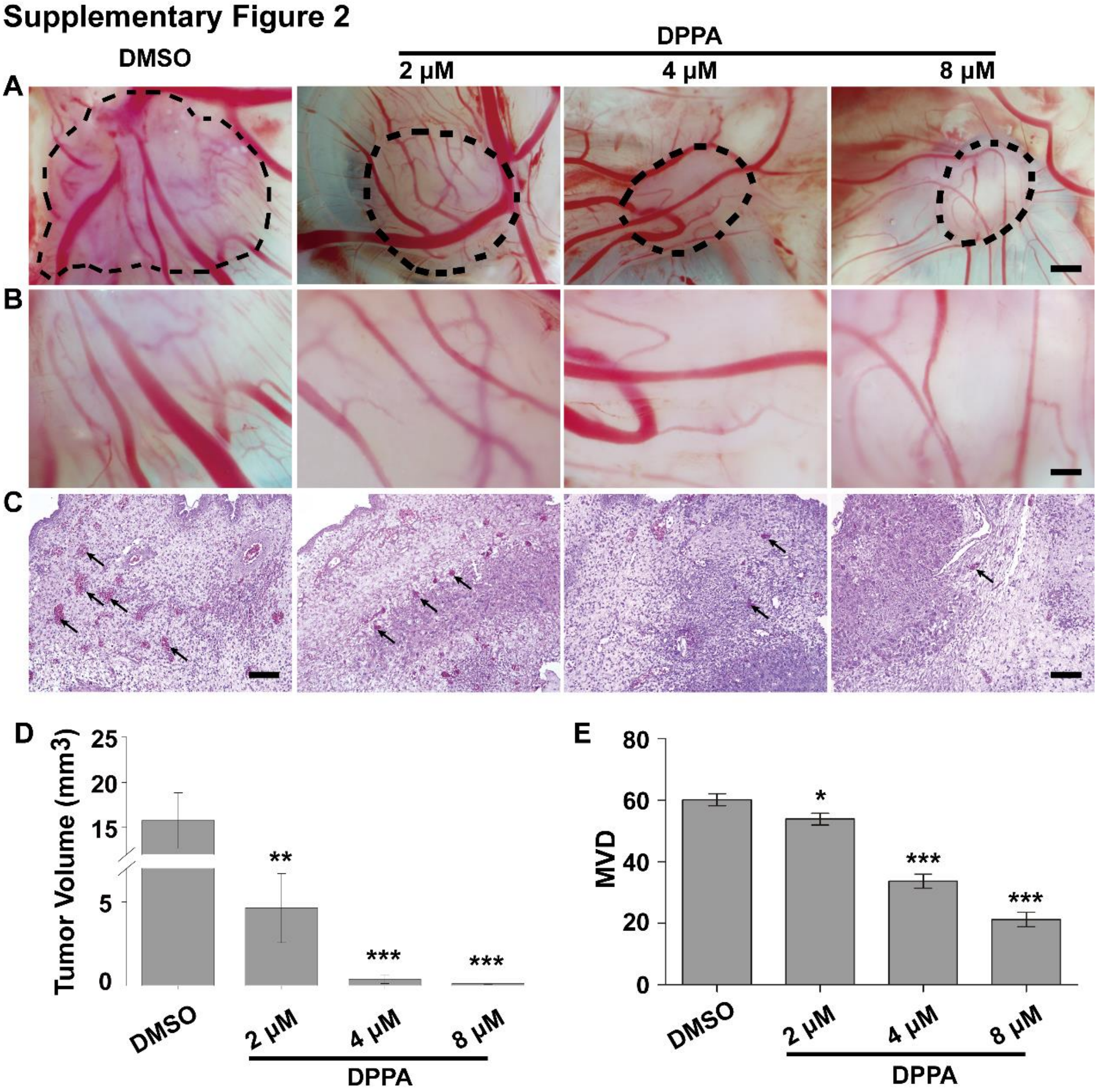

Supplement: Supplementary file 1 [file JCMM-22-4760-s001.pdf]
